# Learning and transfer of complex motor skills in virtual reality: a perspective review

**DOI:** 10.1186/s12984-019-0587-8

**Published:** 2019-10-18

**Authors:** Danielle E. Levac, Meghan E. Huber, Dagmar Sternad

**Affiliations:** 1 Department of Physical Therapy, Movement and Rehabilitation Sciences, Northeastern University, 407c Robinson Hall, 360 Huntington Ave, Boston, MA 02115 USA; 2 Department of Mechanical Engineering, Massachusetts Institute of Technology, 77 Massachusetts Ave, Bldg 3, Rm 143, Cambridge, MA 02139 USA; 3 Biology, Electrical and Computer Engineering, and Physics, Northeastern University, 503 Richards Hall, 360 Huntington Avenue, Boston, MA 02118 USA

**Keywords:** Sensorimotor control, Motor learning, Transfer, Complex skills, Virtual reality, Virtual environments, Rehabilitation, Variability, Redundancy

## Abstract

The development of more effective rehabilitative interventions requires a better understanding of how humans learn and transfer motor skills in real-world contexts. Presently, clinicians design interventions to promote skill learning by relying on evidence from experimental paradigms involving simple tasks, such as reaching for a target. While these tasks facilitate stringent hypothesis testing in laboratory settings, the results may not shed light on performance of more complex real-world skills. In this perspective, we argue that virtual environments (VEs) are flexible, novel platforms to evaluate learning and transfer of complex skills without sacrificing experimental control. Specifically, VEs use models of real-life tasks that afford controlled experimental manipulations to measure and guide behavior with a precision that exceeds the capabilities of physical environments. This paper reviews recent insights from VE paradigms on motor learning into two pressing challenges in rehabilitation research: 1) Which training strategies in VEs promote complex skill learning? and 2) How can transfer of learning from virtual to real environments be enhanced? Defining complex skills by having nested redundancies, we outline findings on the role of movement variability in complex skill acquisition and discuss how VEs can provide novel forms of guidance to enhance learning. We review the evidence for skill transfer from virtual to real environments in typically developing and neurologically-impaired populations with a view to understanding how differences in sensory-motor information may influence learning strategies. We provide actionable suggestions for practicing clinicians and outline broad areas where more research is required. Finally, we conclude that VEs present distinctive experimental platforms to understand complex skill learning that should enable transfer from therapeutic practice to the real world.

## Introduction

The goal of rehabilitation interventions for clients with neurological impairments is to (re)learn motor skills during therapeutic practice and transfer those improvements to functional activities in daily life. Researchers and clinicians seek to understand the content and structure of practice that facilitates such learning and transfer for different tasks, environmental contexts and clinical populations [1]. Although (re)learning activities of daily living is the focus of neurological rehabilitation, much of the evidence base for therapeutic interventions stems from basic or clinical research on simple experimentally-controlled tasks, such as reaching to a target in the horizontal plane or learning a finger tapping sequence. While these simplified tasks are very different from the tasks of daily life, they facilitate precise quantification of performance variables and stringent hypothesis testing, providing insights into basic principles of motor control and learning. However, their deliberately reduced testbeds lack a feature that is pervasive in real-world tasks: the affordance of multiple options to achieve a movement goal [2]. Hence, principles of learning derived from these simple movement paradigms may not translate into useful transfer-oriented principles for rehabilitation [3].

With some exceptions, e.g., Constraint-Induced Movement Therapy [4], few rehabilitation interventions can consistently demonstrate evidence for transfer from practiced tasks to non-treatment contexts. This is also true for the rehabilitation-based use of virtual environments (VEs): computer hardware and software systems that generate simulations of real or imagined environments with which participants interact using their own movements [5]. VEs differ according to viewing medium, level of immersion, and type of interaction [6]. While practice in a variety of VEs offers promising evidence for skill acquisition as compared to conventional interventions in many rehabilitation populations, [e.g. 7–10] the focus has been predominantly on training simplified movements. This may be one reason why successful transfer of skill learning to non-practiced tasks and real-life contexts often remains a challenge [11–16]. As such, the design of both virtual and conventional interventions requires greater understanding of how humans acquire, retain and transfer real-world skills. We propose that VEs themselves can serve as useful experimental platforms to gain this knowledge as they allow the study of these complex skills with sufficient experimental control to draw scientifically tractable conclusions [2].

### Complex real-world tasks have nested redundancy

In the motor learning literature, the adjective “complex” is often treated synonymously with “difficult” [17, 18]. For example, a task can be labelled as difficult or complex when reaction time or movement time are relatively long, when skill improvement requires long hours of practice, or when the task poses high demands on the learner’s attention and memory [3]. To sharpen the discussion, we reserve the term ‘complex’ for tasks with *nested redundancy*. Redundancy is present when there is a greater number of execution variables than variables that define the result of the task. The well-known example for motor redundancy is pointing to a target with one's fingertip, which can be achieved with many different joint configurations, because the arm (without the hand) has 7 degrees of freedom, while the target is defined in 3 degrees of freedom.

**Fig. 1:**
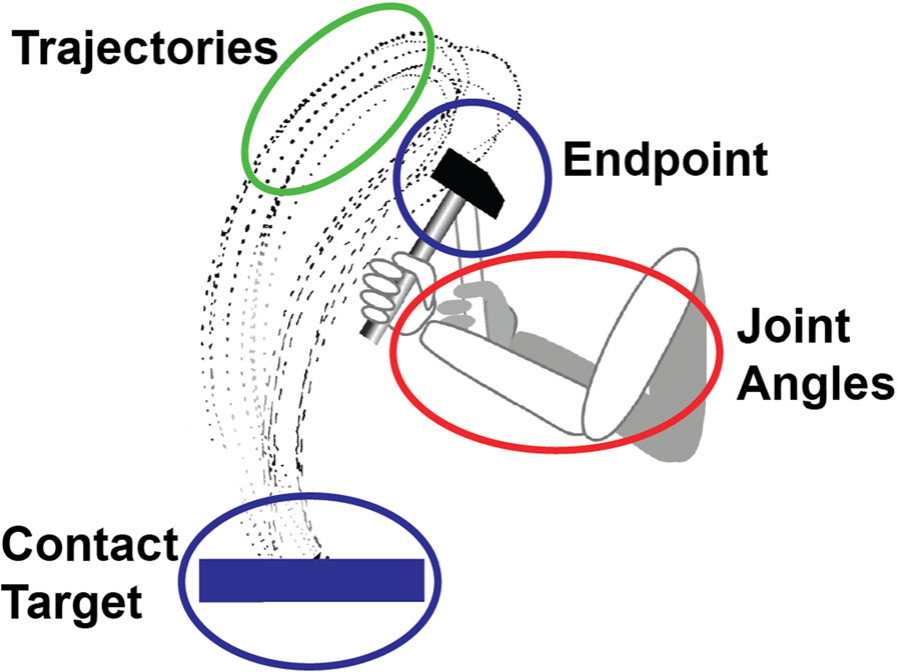
Nested redundancies in a hammering task

However, real-world tasks have another level of redundancy that lies in the task itself. Imagine you are asked to point to a line, where each location on the line is equally correct. Here, the task itself allows an infinite number of “solutions”. And of course, each of those solutions can be achieved with an infinite number of joint configurations. Further, each of the points on the target line can be reached with an infinite number of trajectories from the starting point towards the target line. It is these nested redundancies that characterize the challenge and the richness of real-world tasks. Figure 1 illustrates these nested redundancies with the example of hammering a target on an anvil. The traces are the original recordings of Bernstein from the 1930s, showing the tip of a hammer in the sagittal plane [19]. The added simplified arm with three joints can take on infinite configurations for any position of the hammer endpoint in the 2D plane (intrinsic redundancy). Next, the trajectories of the repeated endpoint actions take on many different shapes, in fact infinitely many shapes, while all of them hit the anvil (extrinsic redundancy). Finally, the anvil or target itself is not a point but a line, where any contact is regarded as a successful hit (task redundancy) [20]. Examples for these nested redundancies are ubiquitous in real life, from combing one’s hair to cutting a steak with a knife and fork. Performers must choose (implicitly or explicitly) from an infinite range of possible solutions, each leading to successful task accomplishment [2]. We define such actions as ‘complex’ skills. To gain insight into these ever-present control challenges and opportunities, scientific inquiry must move beyond simple tasks where redundancy has been purposefully removed and begin to examine more complex tasks.

### Using virtual environments to overcome the challenges of studying complex skills

Studying how humans both manage and exploit redundancy necessitates research on platforms that can support complexity without sacrificing experimental control. However, the study of complex real-world skill learning is stymied by the inherent difficulty in controlling and accurately measuring all the relevant human- and task-related features. For example, in grasping a cup and leading it to one’s mouth to drink, it is important to consider features such as the curvature of the handle and the shape and mass of the cup, as these factors can influence grasp and transport movements. VEs enable such studies because they permit experimenters to control the physics of an object so that it can be rendered and confined to exactly the variables and parameters under analysis [21, 22]. This leaves no uncontrolled aspects as would occur in real-life tasks [2]. Precise knowledge of the object eliminates inaccuracies that can arise from simplifying assumptions about. These attributes facilitate evaluation of how performers deal with redundancy and learn optimal task solutions. Table 1 provides examples of how virtual tasks can present a versatile platform for theoretically-grounded, quantitative assessment and guidance of complex skill learning.

**Table 1:** Attributes of virtual environments that facilitate the study of complex skill learning and transfer

Attributes of virtual environments	Examples
Detailed measurements of execution (or process) beyond course-grained descriptive outcome measures of motor performance.	Precise tracking of human kinematics and interaction with virtual objects. Ability to combine measurement of task execution variables and result variables.
Ability to mathematically model motor tasks and vary relevant task parameters	Mathematical modeling of task physics makes explicit the variables that define task execution and result. Parameters that can be manipulated include those that reduce or augment task error. Such task constraints can be systematically varied to identify their effect on performance.
Precise simulation of the physics of virtual objects limits uncontrolled aspects that may confound results.	Modeling in a VE confines the task to the measured variables, excluding, for example, environmental noise such as drag or lateral forces influencing the trajectory of a thrown ball.
Ability to examine a range of perceptual conditions with robust experimental control.	VEs enable precise manipulation of experimental parameters, including amount of haptic, haptic, visual or auditory feedback and task difficulty (e.g. changing the size or position of a target), to test hypotheses about performance strategies.

The purpose of this perspective review is to describe recent progress in motor learning research with VE platforms related to two pressing questions in rehabilitation science: 1) Which training strategies in VEs promote complex skill learning? and 2) How can transfer of learning from virtual to real environments be enhanced? These findings are synthesized to provide actionable suggestions for clinicians and highlight areas where future research is needed.

## Methods

Literature selection for the review was conducted in the indexed databases PubMed, IEEE and CINAHL. The search strategy used the keywords ‘redundancy’ OR ‘complex*’ AND ‘task’ OR ‘skill’ AND ‘motor learning’ OR ‘transfer’ AND ‘virtual reality’ OR ‘virtual environment’ (for Question 1). For Question 2, we used ‘virtual reality’ OR ‘virtual environment’ AND ‘motor learning’ OR ‘transfer’ OR ‘generalization’ AND ‘rehabilitation’ OR ‘physical therapy’ OR ‘physiotherapy’ OR ‘occupational therapy’. Our review includes experimental studies published since the year 2000, including our own work related to the two research questions. Methodological quality of the included studies was not evaluated. Studies that described clinical trials and interventions evaluating the effectiveness of VEs were not included as our interest was in experimental studies exploring mechanisms of learning and transfer, rather than in the efficacy of VE intervention programs. Our search yielded 46 studies, listed in Table 2.

**Table 2:** Studies included in the review, listed in the sequence they are referenced

Focus	Title	Authors	Year	Population
*Understanding variability in complex skill learning*	Acquisition of novel and complex motor skills: stable solutions where intrinsic noise matters less.	Sternad D, Huber ME, Kuznetsov N	2014	Unimpaired
	From theoretical analysis to clinical assessment and intervention: Three interactive motor skills in a virtual environment.	Sternad D	2015	Unimpaired, impaired
	Exploiting the geometry of the solution space to reduce sensitivity to neuromotor noise.	Zhang Z, Guo D, Huber ME, Park SW, Sternad D	2018	Unimpaired
	State space analysis of timing: exploiting task redundancy to reduce sensitivity to timing.	Cohen RG, Sternad D	2012	
	Bouncing a ball: tuning into dynamic stability.	Sternad D, Duarte M, Katsumata H, Schaal S	2001	
	One-handed juggling: A dynamical approach to a rhythmic task	Schaal S, Atkeson CG, Sternad D	1996	
	Passive stability and active control in a rhythmic task.	Wei K, Dijkstra TM, Sternad D	2007	
	Human control of interactions with objects: Variability, stability and predictability.	Sternad D	2017	
	The influence of movement initiation deficits on the quantification of retention in Parkinson’s disease.	Pendt LK, Maurer H, Müller H.	2012	Impaired
	Healthy and dystonic children compensate for changes in motor variability.	Chu VW, Sternad D, Sanger TD	2013	Unimpaired, impaired
*Inducing variability to enhance learning*	Motor learning through induced variability at the task goal and execution redundancy levels.	Ranganathan R, Newell KM	2010	Unimpaired
	Emergent flexibility in motor learning.	Ranganathan R, Newell KM	2010	
	Changing up the routine: intervention-induced variability in motor learning.	Ranganathan R, Newell KM	2013	
	High variability impairs motor learning regardless of whether it affects task performance.	Cardis M, Casadio M, Ranganathan R.	2018	
	Directionality in distribution and temporal structure of variability in skill acquisition.	Abe MO, Sternad D	2013	
	Learning a throwing task is associated with differential changes in the use of motor abundance.	Yang JF, Scholz JP	2013	
*Amplification of visual errors to stimulate learning*	Using noise to shape motor learning.	Thorp EB, Kording KP, Mussa-Ivaldi FA	2017	
	Neuromotor noise is malleable by amplifying perceived errors.	Hasson CJ, Zhang Z, Abe MO, Sternad D	2016	
	Persistence of reduced neuromotor noise in long-term motor skill learning.	Huber ME, Kuznetsov N, Sternad D	2016	
	Visual error augmentation enhances learning in three dimensions.	Sharp I, Huang F, Patton J	2011	
	Visuomotor discordance during visually-guided hand movement in virtual reality modulates sensorimotor cortical activity in healthy and hemiparetic subjects.	Tunik E, Saleh S, Adamovich SV	2013	Unimpaired, impaired
	Visuomotor gain distortion alters online motor performance and enhances primary motor cortex excitability in patients with stroke.	Bagce HF, Saleh S, Adamovich SV, Tunik E	2012	
	Visuomotor discordance in virtual reality: effects on online motor control.	Bagce HF, Saleh S, Adamovich SV, Tunik E	2011	
	Effect of error augmentation on brain activation and motor learning of a complex locomotor task.	Marchal-Crespo L, Michels L, Jaeger L, Lopez-Oloriz J, Riener R	2017	Unimpaired
	Haptic error modulation outperforms visual error amplification when learning a modified gait pattern.	Marchal-Crespo L, Tsangaridis P, Obwegeser D, Maggioni S, Riener R	2019	
*Manipulation of task physics for implicit guidance*	Implicit guidance to stable performance in a rhythmic perceptual-motor skill.	Huber ME, Sternad D	2015	
*Inconclusive evidence of skill transfer from virtual to real environments*	Functional performance comparison between real and virtual tasks in older adults: A cross-sectional study.	Bezerra IMP, Crocetta TB, Massetti T, Silva TDD, Guarnieri R, Meira CM, et al.	2018	
	Transfer of motor learning from virtual to natural environments in individuals with cerebral palsy.	de Mello Monteiro CB, Massetti T, da Silva TD, van der Kamp J, de Abreu LC, Leone C, et al.	2014	Impaired
	Motor learning from virtual reality to natural environments in individuals with Duchenne muscular dystrophy.	Quadrado VH, Silva TDD, Favero FM, Tonks J, Massetti T, Monteiro CBM.	2017	
	Achievement of virtual and real objects using a short-term motor learning protocol in people with Duchenne muscular dystrophy: A crossover randomized controlled trial.	Massetti T, Favero FM, Menezes LDC, Alvarez MPB, Crocetta TB, Guarnieri R, et al.	2018	
	Transfer of a skilled motor learning task between virtual and conventional environments.	Anglin J, Saldana D, Schmiesing A, Liew S.	2017	Unimpaired
	Is children’s motor learning of a postural reaching task enhanced by practice in a virtual environment?	Levac DE, Jovanovic B.	2017	
*Differences in movement kinematics between virtual and real environments*	Upper limb kinematics in stroke and healthy controls using target-to-target task in virtual reality.	Hussain N, Alt Murphy M, Sunnerhagen KS	2018	Unimpaired, impaired
	Kinematics of reaching movements in a 2-D virtual environment in adults with and without stroke.	Liebermann DG, Berman S, Weiss PLT, Levin MF	2012	
	Effects of real-world versus virtual environments on joint excursions in full-body reaching tasks.	Thomas JS, France CR, Leitkam ST, Applegate ME, Pidcoe PE, Walkowski S.	2016	Unimpaired
	Viewing medium affects arm motor performance in 3D virtual environments.	Subramanian SK, Levin MF.	2011	
	Validation of reaching in a virtual environment in typically developing children and children with mild unilateral cerebral palsy.	Robert MT, Levin MF	2018	Unimpaired, impaired
	Comparison of grasping movements made by healthy subjects in a 3-dimensional immersive virtual versus physical environment.	Magdalon EC, Michaelsen SM, Quevedo AA, Levin MF	2011	Unimpaired
	Planning and adjustments for the control of reach extent in a virtual environment.	Stewart JC, Gordon J, Winstein CJ.	2013	
	Quality of grasping and the role of haptics in a 3-D immersive virtual reality environment in individuals with stroke.	Levin MF, Magdalon EC, Michaelsen SM, Quevedo AAF	2015	Unimpaired, impaired
*Differences in learning mechanisms in virtual and real environments*	Visuomotor adaptation in head-mounted virtual reality versus conventional training.	Anglin JM, Sugiyama T, Liew SL	2017	Unimpaired
*Enhancing task transfer through VE fidelity and dimensionality*	Goal-related feedback guides motor exploration and redundancy resolution in human motor skill acquisition.	Rohde M, Narioka K, Steil JJ, Klein LK, Ernst MO	2019	
	Learning redundant motor tasks with and without overlapping dimensions: facilitation and interference effects.	Ranganathan R, Wieser J, Mosier KM, Mussa-Ivaldi FA, Scheidt RA	2014	

### Question 1: which training strategies in virtual environments promote complex skill learning?

**Fig. 2:**
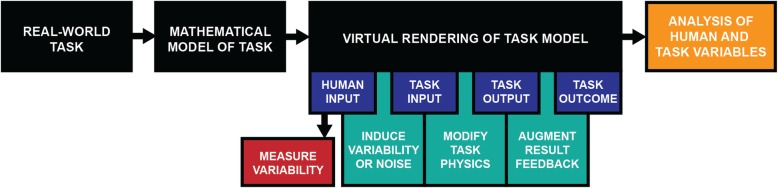
Data acquisition, measurements and experimental manipulations in virtual rendering of real-life tasks. Overview of how a real-world task is implemented in a virtual environment to afford manipulation of task variables and fine-grained analysis of human behavior. To begin, a real-world task requires to a mathematical model in order to be implemented in a virtual environment. This model necessarily reduces the full complexity of the real behavior into task variables that are of interest. After the task is virtually rendered, the human interactive input can be measured, including its variability. However, the virtual rendering also allows to induce additional variability. Further, it can modify the task physics and provide augmented feedback about the result

To answer this question, we reviewed studies exploring how modeling and modifying task attributes in VEs enables new perspectives on complex skill learning and supports novel forms of feedback and guidance. Figure 2 overviews the process and possibilities for data acquisition, measurements and experimental manipulations in virtual rendering of real-life tasks.

#### Understanding variability in complex skill learning

Reviews of research on skill acquisition (e.g. [20, 23]) highlight how skill improvement is achieved by reducing, processing and exploiting variability. To clarify terminology, variability is defined as an umbrella term “for all sets or series of observations that are non-constant and … non-stationary.” [20] Variability in motor output can be caused by stochastic processes or intrinsic noise manifested as a lack of temporal or spatial structure. In addition, variability can also be a positive feature, serving as active exploration for information gathering. In that case, variability can have structure in time series and distributions that is informative both for the performer and the scientist. Reducing the stochastic element of variability is certainly important for simple tasks without redundancy, where success is limited by how much actors can control and reduce the inherent variability in their neuromotor system. However, some amount of variability or noise always remains, even when healthy individuals repeat the same movement pattern under fixed and well-learned conditions [23, 24]. In complex tasks with nested redundancy, variability can be present without detrimental effects on the task outcome; variability in the motor output is therefore a window into understanding processes of learning and exploration. As such, it is important to examine how variability evolves in skill learning to understand how and when to assist performers in the search for effective solutions.

A first example of how variability is more than simple nuisance comes from our own work. In a series of studies Sternad and colleagues developed a virtual throwing task that has the essential redundancy with a manifold of solutions [2, 21, 25]. The learner throws a ball tethered to a post by a pendulum-like string, with the goal to hit the target on the opposite side of the pole. Two variables in execution, position and velocity at ball release, map into one result variable, error from hitting the target. This mapping from two variables to one variable allows for different combinations of the two execution variables that all lead to the same result variable, error. The set of position and velocity combinations that achieve zero error defines the solution manifold which contains a mathematically infinite number of executions. Knowledge of this solution manifold affords the analysis of variability in a tractable way [2].

When examining distributions of ball releases over practice time, the analysis distinguished between three different components of variability that contributed to performance improvement. “Tolerance” denotes the component that evaluates how close the data are to the most noise-tolerant region in the solution space; tolerance to noise is defined as the area in solution space where noise and perturbations have less effect on overall performance. “Covariation” is a component that evaluates how the data differ from a normal distribution and align with the solution manifold. “Noise” is the random component in the data set [24, 26]. “Tolerance” can be interpreted as a quantification of exploratory processes, while “Covariation” can be likened to an active process of improving the probability of success. A series of experiments showed that although participants decreased their overall variability with practice, reducing random noise was not the dominant avenue for improving performance. Rather, people first developed strategies that optimized “Tolerance”; subsequently, they reduced “Noise” and then targeted “Covariation” by exploiting the structure of the solution manifold [2, 24]. In a complementary set of studies, the arm trajectories were analyzed to reveal that with practice they aligned with the solution manifold [25, 27]. These strategies enable success in the face of intrinsic neuromuscular noise.

A second task by Sternad and colleagues used a real racket to rhythmically bounce a virtual ball to a virtual target. Again, this task was modeled as an extremely simple physical system: a horizontal racket contacting a ball, where both racket and ball are confined to the vertical direction [28, 29]. The task has redundancy as different ball-racket contacts can propel the ball to the same target height: racket and ball velocity at contact and the contact position with respect to the target height determine the result, i.e., three variables map into one [30, 31]. In addition, the task is a dynamic system: impacts between the ball and the racket occur in a rhythmic sequence and the characteristics of one bounce influence the next bounce. Specifically, the ball velocity at contact is determined by the previous bounce; this differs from the discrete ball throwing task where successive throws are separated by rest and are in principle independent. Mathematical analyses established that the task affords a dynamically stable solution obtained when the racket hits the ball in its upwardly decelerating phase [28, 29]. This demonstrates that enhancing task success can be achieved not only by reducing variability in task execution, but also by achieving dynamically stable solutions. A series of experiments demonstrated that, indeed, performers discovered the solution that exploited task stability and, concomitantly, decreased variability [32, 33]. When facing perturbations, performers explored the solution space and found new solutions. Notably, in these new solutions, neither mean performance nor the remaining variability were affected by the perturbation [31]. This suggests that performers were aware of their variability.

These studies demonstrate that using a VE where the space of all solutions is known facilitates understanding of how performers explore and find solutions within the available redundancy. Overall, the throwing and ball bouncing studies exemplify how a complex real-world task can be simplified and modeled in a VE without sacrificing the essential redundancy. They also illustrate how the virtual rendering affords measurement and quantitative understanding of the structure of variability and thereby enables new ways to describe stages of learning [2, 21].

#### Inducing variability to enhance learning

A subsequent avenue of research examined how manipulation of variability may enhance motor learning. Ranganathan et al. [34, 35] evaluated the benefits of inducing variability using a targeting task on a digitizing tablet. The exact trajectory to reach the target was not prescribed and therefore the task presented redundancy. When performers were induced to explore multiple trajectories (to increase their movement variability), their performance was less successful than when they focused on the most task-relevant parameters (in this case, on the location of a moving target) [34–36]. This unexpected result may be due to the undemanding nature of this targeting task or due to the low fidelity of the set-up [34].

In a follow-up study, Ranganathan and colleagues [37] evaluated the impact of external perturbations to add variability in movement execution, and used a more challenging and more immersive virtual shuffleboard task. Participants moved two manipulanda to slide a virtual puck towards a target; the velocity of the puck at release (the sum of the two manipulanda’s velocities) fully determined the puck’s distance; as such, reaching the target was possible via an infinite number of combinations of the two hands’ velocities. Different viscous fields were induced via the manipulanda with the expectation that the resultant variability would enhance exploration of the solution space and thereby improve subjects’ performance. Specifically, variability was induced in null space (i.e., along the solution manifold) and in task space, with the prediction that only variability in task space would affect performance. However, while all participants reduced their errors with practice, the type of perturbation did not have the expected influence [38, 39]. Additionally, larger perturbations had detrimental effects not only on performance but also on retention and transfer [37]. These results demonstrated that while externally-induced perturbations may increase variability, the nature of this variability is very different from the internally-produced variability that may benefit motor learning.

In contrast, a study by Thorp et al. [40] found that inducing variability via adding external noise on select dimensions of the task can indeed have beneficial effects on transfer. Noise was added during a bimanual task with a cursor and targets displayed in a VE. Participants grasped inertial measurement units and learned to control a cursor in the VE, mapping 4 dimensions (pitch and roll of each sensor) to the 2-dimensional cursor movement. Participants moved the cursor to intercept different targets in the VE; in the noise condition, artificial signal noise was added to select dimensions of the mapping to evaluate whether participants would learn to minimize noise or find alternative strategies to cope with the noise [40]. With practice, participants not only learnt a noise-tolerant strategy, but also better transferred their learning to new target locations. These findings demonstrate that the imposed noise could indeed guide participants to explore the null space. This exploration may also have prepared them for transfer to subsequent unpracticed versions of the task [40].

Overall, the reviewed experimental manipulations demonstrate a range of promising and less-promising options; more work is required to evaluate the effects of practice conditions that elicit trial-to-trial variability before conclusions can be made about the effectiveness of this training strategy in VEs.

#### Amplification of visual errors to stimulate learning

While VEs can implement conventional ways of providing explicit feedback about performance and results, they also afford a variety of possibilities that are not available in real-world settings. One such example is error amplification [41–43]. While physical or haptic error amplification (and reduction) requires the use of robotic interfaces [44–46], visual error amplification or distortion can be presented in a VE without the use of a robot. For example, Hasson et al. [43] used the virtual throwing task earlier described to explore the effect of visual error amplification after participants had reached a performance plateau following 3 days of practice. While performance in the control group stagnated, error amplification in the experimental group led to further improvement in performance. These results held for both stochastic and deterministic error amplification. Further decomposition of the variability in the sequence of trials showed that it was the random noise that subjects reduced, indicating the potential of this intervention for rehabilitation. A complementary study by Huber et al. manipulated the perceived error by changing the threshold for signaling success [47]. After initial practice with a given threshold, the experimental group experienced an elevated threshold and had to perform better to receive a success signal. As anticipated, they improved their performance. In addition, when the success feedback returned to the initial level, the improved performance persisted for five more days. These encouraging results are consistent with those of Sharp et al. [42], who used error augmentation in a targeted reaching task in a VE. Subjects who trained under this error augmentation significantly improved their performance, and this difference persisted upon removal of the augmentation [42]. These results are clearly encouraging for therapeutic purposes.

From a rehabilitation perspective, related studies of error augmentation in the form of induced visuomotor discordances have explored how such practice conditions can trigger functional neuroplasticity after injury [48–50]. For example, participants with stroke demonstrated increased activation of the ipsilesional motor cortex during discordant feedback conditions, indicating that this strategy may be useful within VE-based training designed to facilitate motor recovery in the affected hand [48].

Visual error augmentation has also been explored in VE-based lower-extremity tasks, although with less success. Marchal-Crespo et al. [51] explored the effect of error augmentation when healthy participants learned a dual-leg coordination pattern to track an ellipse presented in the VE. While amplifying errors enhanced skill acquisition in participants who initially demonstrated greater skill, it negatively impacted transfer due to a slightly different coordination strategy [51]. The same group of researchers evaluated the effect of haptic error versus visual error amplification in a VE [52]. Participants who trained a novel asymmetric gait pattern with visual error amplification demonstrated poorer transfer to a free walking condition as compared to the haptic perturbation group. Given these negative findings with regards to transfer, more evidence is required that the positive effects can persist and transfer into real-world settings to solidify the rehabilitation potential of visual error amplification.

#### Manipulation of task physics for implicit behavioral guidance

A lesser-explored option for feedback provision in VEs is manipulating the physics of the task to target implicit learning mechanisms. Rather than providing explicit instructions for performance or knowledge of results, VEs can guide learners implicitly, without providing declarative knowledge about how to perform the task. Such implicit guidance has potential advantages for learning in rehabilitation populations, because it allows for development of procedural skill that does not rely on working memory mechanisms [53, 54]. For example, returning to the virtual ball bouncing task earlier described, Huber et al. [55] aimed to implicitly steer learners towards the desired solution of rhythmically bouncing the ball with dynamic stability. As mentioned, dynamic stability is desirable as it obviates the need for corrections, since they die out by themselves. Previous mathematical analyses showed that dynamic stability depended on the racket acceleration at ball contact, specifically, a decelerating racket trajectory at ball contact. Experimental results showed that practice was needed to find these strategies. Hence, this study modified the ball-racket contact by adding a time delay to the racket velocity at contact to induce participants to contact the ball later in their racket trajectory. The experimental group indeed adopted dynamically stable solutions earlier than the control group. Importantly, and in contrast to typical adaptation experiments where the adapted behavior returned to baseline within a few trials, these solutions persisted even after the guidance was removed [55]. Although this manipulation modifies the task physics and induces changes in the trajectories which is not as straightforward as error augmentation, it holds promise as an alternative route to guide learners towards a desired solution. Most importantly, modifications during practice need to persist after removal of the manipulation, which has not been achieved in typical adaptation paradigms.

#### Insights for rehabilitation

The reviewed studies explored ways to observe and manipulate variability in VEs with the goal of identifying implications for therapeutic practice. Findings align with the reflections of Harbourne and Stergiou [56, 57] who encourage therapists to think differently about human movement variability in rehabilitation. They suggest moving away from a focus on limiting variations to achieve consistent and successful performance towards emphasizing variations in task performance that ultimately achieve more adaptability. Similarly, Orth and colleagues [58] argue that movement variability stemming from individual and task constraints allows learners to find creative solutions in response to movement problems. Building on the results reviewed above, therapists can help patients search for solutions that are more stable with respect to their own inherent variability; specifically, they can assist them to ‘improve’ rather than reduce their variability. We have discussed how knowledge of the task can aid in decomposing the components of variability with respect to the solution manifold and parsing out the unstructured intrinsic noise that can be detrimental. In the effort to reduce this detrimental noise, therapists can guide clients to ‘channel’ their variability to have minimal impact on task performance. Pragmatically, this means guiding learners towards more ‘noise-tolerant’ solutions that support flexibility and adaptations to perturbations. To achieve this objective, therapists may reflect on the metrics they use to measure the effectiveness of their interventions, moving beyond simple measures of task success to more execution-oriented metrics.

A therapeutic example is when the therapist encourages variability by asking clients to practice standing up from chairs of different heights and shapes, and from seats with or without armrests. While such training is certainly important, it may also be relevant to encourage clients to discover the best solutions among the numerous options in how to accomplish a single outcome; in this case, exploring different methods to stand up from a chair of a specific height or shape [56]. This is particularly relevant for individuals with constraints due to neurological impairment, [35] who may have fewer movement options, and for whom the resulting repetitive and compensatory movements may ultimately lead to musculoskeletal deterioration. Emphasizing variability in movement execution differs from approaches based on neurodevelopmental or neuromaturational theories of motor learning which encourage the client to perform the task in a consistent manner that is presumed to be biomechanically correct. Indeed, such training in consistency may limit the ability to discover solutions among the multiple options suited for a specific person and context. However, the benefits of explicitly training a variety of movement executions to explore or exploit available redundancy, as well as strategies for inducing variability, require further evaluation, as does their impact on retention and transfer [36].

#### Next steps for research

As most of the work to date has focused on healthy populations, its application to understanding differences in skill learning in neurologically-impaired populations must be investigated. For example, Pendt et al. [59] have used the throwing task in adults with Parkinson Disease (PD). Older adults with PD were able to improve and retain the skill with practice, yet experienced more warm-up decrements than did healthy controls, which ultimately led to less improvement. In a study on children with dystonia, Sternad and colleagues attenuated the subjects; intrinsic variability that was visually presented. This enabled children to improve their strategies as they were no longer confounded with their high intrinsic noise [60]. This study showed that children with dystonia could achieve control over their movements and adapt their behavior when they could see their behavior without the excessive noise. These results underscore the importance of exploring the role of movement variability in populations with altered kinematic systems and decreased intrinsic redundancy, such as patients with stroke [61].

Harnessing the potential of VEs for clinical assessment via fine-grained quantitative measurement of complex skills is another important avenue for further development. Unlike traditional rating scales, VE platforms can assess how specific motor impairments limit movement strategies in different task configurations. Finally, therapists can take advantage of the fact that a VE can implement any task physics, even dynamics that defy the laws of Newtonian physics, to devise novel task characteristics and subsequently develop new interventions to guide learning and transfer. The possibilities are limitless.

### Question 2: how can transfer of learning from virtual to real environments be enhanced?

The acclaim of VEs for rehabilitation stems from their potential to obtain and exploit evidence-based insights for motor learning. Advantages are many, including that VEs can provide abundant practice repetitions, deliver multi-sensory feedback, individualize challenge, and engage and motivate users with salient, enriched environments [62–64]. In addition, VEs afford detailed measurement options and cost-saving potential for home-based tele-rehabilitation [65, 66]. There is indeed already a promising body of evidence for effective VE-based interventions in populations such as stroke, [8] multiple sclerosis, [9] Parkinson, [10] and cerebral palsy (CP) [7]. However, this promise is handicapped by inconclusive demonstration that the acquired skills from VE practice can be transferred to the real-world [12, 13, 15, 16].

#### Inconclusive evidence of skill transfer from virtual to real environments

A relatively small number of studies have explored motor skill acquisition and transfer from virtual to real environments in healthy and neurologically impaired populations. Several studies used a simple coincidence timing task in which participants intercepted a falling virtual object by either pressing a key on computer (physical task) or making a hand movement tracked by webcam (virtual task) [67–69]. In adolescents with CP, older adults and healthy controls, practice in the VE did not transfer to improved performance of the real-world task [68, 69]. The authors suggest that the internal model for the task could not generalize because of different sensory-motor information and spatiotemporal organization between the virtual and real interfaces. Specifically, the lack of haptic input in the VE task forced participants to rely on visual information alone, leading to different perceptual-motor couplings than in the real task. In contrast, individuals with Duchenne Muscular Dystrophy (DMD) did demonstrate transfer of this task from the virtual to the real environment [69]. Quadrado and colleagues attributed this finding to the fact that the VE task was motorically more challenging, suggesting that transfer may be enhanced by purposefully increasing task difficulty in VEs [69]. However, this speculation is inconsistent with the negative finding by Massetti et al. [70] in which individuals with DMD showed no transfer from a virtual to a real environment in a reaching task. Another study with healthy young adults, where participants learned a sequential visual isometric pinch task either with a head-mounted display (HMD) or in a conventional environment, showed that those who trained in the HMD did not transfer the task to its real-life version [71]. Instead, their performance degraded in this environment, despite identical task interaction in both environments. Finally, in a typically developing pediatric sample, Levac and Jovanovic [72] compared a novel postural reach-to-touch skill in either a flat-screen projection VE or a real environment. The results showed that children who acquired the skill in the VE could not transfer performance to the real environment. The authors suggest that unique task demands in each environment - in particular, the lower demands on target hit precision due to lack of haptic and depth cues in the VE - influenced how, and what, skill was acquired.

**Fig. 3:**
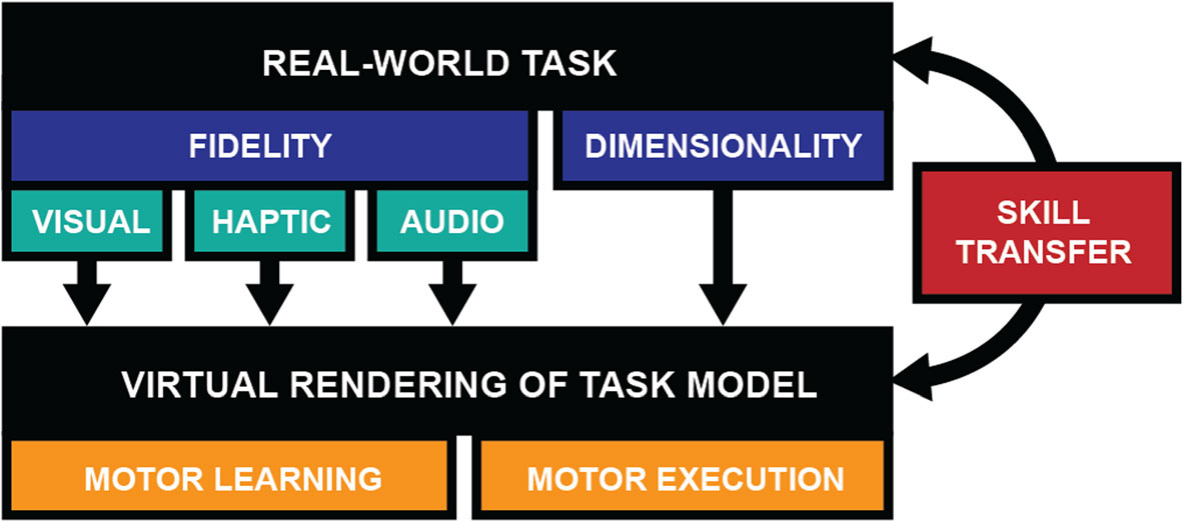
Overview of aspects that affect the success of the virtual rendering of real-world tasks and the transfer of skills from the virtual to the real world. Fidelity and dimensionality of the virtual environment determines motor learning, motor execution and, as a result, skill transfer. A virtual environment affords the study of execution and learning of motor skills with the goal of enabling transfer to real-world activities

Discrepancies in transfer success as reported in these studies may in part be ascribed to several methodological shortcomings, such as small sample sizes, low practice dosages, and short retention intervals. However, they may also be real and due to the differing sensory-motor information between virtual and real environments. In the following section, we summarize the reported differences in motor execution and motor learning between VEs and real-world environments and discuss how they may impact skill transfer. Moreover, we discuss methods of enhancing skill transfer by increasing practice similarity between virtual and real tasks. Figure 3 overviews how the fidelity and dimensionality of the virtual environment determines motor learning, execution and, as a result, skill transfer.

#### Differences in movement kinematics between virtual and real environments

Examining the similarity of directly-tracked movements in virtual and real environments can shed light on the effects of different viewing mediums in the VE on transfer of skill to real-world scenarios. Kinematic differences in unconstrained, goal-directed reaching between 2D flat-screen displays, 3D head-mounted displays (HMDs) and the real environment have been explored in adults with stroke, adults with DMD, children with CP and typically developing controls [73–79]. Comparisons of reaching movements in a HMD versus in a real environment in healthy adults and adults post-stroke have shown that movements in the HMD were slower and had different spatial and temporal kinematics [73, 78, 80]. These differences were attributed to the uncertainty of object location in the VE [73, 78, 80]. Subramanian and Levin [76] found that subjects with and without stroke made more movement errors and had slower movements in a HMD compared to a flat-screen projection system. However, Campbell and Stewart [79] reported that reaching movements in non-disabled adults in a HMD did not differ from reach kinematics in the real world. In VEs with 2D flat-screen displays, studies in adults with and without stroke [74] and in children with CP [77] showed decreased movement quality in the VE as compared to the real environment. A limitation of these studies was their focus on simple reaching tasks that did not have to be learnt as they were already in participants’ repertoires. Exploring ‘de novo’ acquisition of complex tasks between viewing mediums may shed further light on whether the visual display influences motor commands in an interaction-specific way, with a potential limitation for transfer.

#### Differences in learning mechanisms in virtual and real environments

Another important question for understanding transfer is the nature of the learning process. Anglin et al. [81] evaluated differences in motor learning mechanisms in a visuomotor adaptation task with a HMD as compared to a conventional environment in healthy subjects. When adapting to the visuomotor rotation, participants altered their motor behavior in response to an external perturbation of the visual information. Visuomotor adaptation occurs via either explicit cognitive strategies or implicitly in which participants are unaware of their strategies. The hypothesis was that the unique experience of the HMD condition would increase participants’ attention and engagement to the task, favoring explicit cognitive strategies, assessed by subsequent self-report. Results were consistent with this hypothesis, although participants in both conditions required the same time to adapt to the perturbation and reduce their errors. Findings suggest that differences in the mechanisms of learning between VE and real environments should be explored in other types of tasks, with consideration of differing task characteristics and the participant’s level of VE experience. The impact of task characteristics is particularly relevant when we consider that VEs are inherently safe environments; as such, practice in a VE might invite more risk and exploration strategies as compared to the same task in the real world [82].

#### Enhancing task transfer through VE fidelity and dimensionality

The hypothesis of specificity of practice expects that transfer is enhanced when therapeutic practice simulates the conditions of real-life performance as closely as possible [83]. There are clear differences in perceptuo-motor affordances and somatosensory information between object interaction in flat-screen VEs, stereoscopic 3D HMD VEs, and the real-world [84, 85]. Critical is the lack of haptic information about interaction forces with virtual objects in a VE. This significant difference in sensory information limits the specificity of task rendering. This raises the question of what degree of task specificity between a VE and the real world is required to enable transfer? [86] One way to address this issue involves understanding VE fidelity: the precision with which a VE imitates interactions in the natural environment [87]. Fidelity can be achieved by the display device and by the interaction methods.

With respect to the display device, HMDs have an advantage for fidelity as they provide a completely simulated experience in which the user’s view of the virtual world changes in accordance with his/her head movements. These display modalities have stereoscopic rendering that preserves depth cues to assist in determining target distance, thereby enabling higher fidelity as compared to a flat-screen that presents 3D computer graphics [84]. Commercially available options include the HTC Vive (HTC Corporation, Taoyuan City, Taiwan) and the Oculus Rift (Oculus VR, Irvine, CA). With respect to interaction methods, systems that include treadmills or 6 DoF motion bases, such as in the Computer Assisted Rehabilitation Environment (CAREN; Motekforce Link, The Netherlands), elicit higher fidelity interactions that mimic real life situations. VEs in which sensor gloves provide haptic feedback that enables users to obtain sensory feedback from virtual object touch reduces the discrepancy between the VE and the physical environment, although the sensory information may not be identical to interaction with a real object [88]. Indirect measurement methods, e.g., through tracking a controller, or direct body tracking, e.g., via the Kinect sensor (Microsoft, Redmond, US), do not necessarily have low fidelity. These interaction methods can elicit movements similar to real-world actions, such as the arm motions required to serve a volleyball in Xbox 360 Kinect Sports game. Indirect movement tracking using controllers such as the Nintendo Wiimote provides greater potential for ‘cheating’ and elicits large variations in movement patterns within and between users [89]. More research is required to explore the relationship between display device, interaction method fidelity and transfer outcomes.

Another index of task specificity that may be relevant to ensure transfer is dimensional matching. This is defined as the accuracy with which interaction methods in VEs replicate control dimensions of the real-world task [90]. VEs with inadequate dimensional matching to the real world have either fewer control dimensions (e.g., not being able to rotate a hand-held virtual object) or too many control dimensions (e.g. a virtual steering wheel with more than one degree of freedom) [90]. Interaction with virtual objects that are displayed on a 2D flat-screen has inherently fewer dimensions, as these objects have only two (x-y) coordinates [87]. Ranganathan et al. [91] explored the importance of shared task dimensions in VEs to evaluate whether this fact influenced transfer between two complex tasks with redundancy. Subjects wore a data glove and practiced 3D finger movements that were displayed in a VE. Subjects learned two tasks that required the same or different configurations for a target in the x-y screen dimensions. Results demonstrated that transfer was facilitated when the two tasks were dimensionally similar. The authors concluded that the similarity (or lack thereof) of known task space dimensions to new tasks can bias exploration and performance during new task acquisition [91].

In summary, the reviewed studies highlight the potential influence of VE fidelity and interaction characteristics on the extent of transfer from virtual to real environments. Essential differences between movement in virtual environments and real-world actions may impact learning strategies and movement quality. However, these differences should in no way negate the potential for VEs as rehabilitation training environments, but rather spur greater inquiry into VE task-specificity to guide transfer-oriented clinical implementation.

#### Insights for rehabilitation

VE-based practice can offer multiple benefits for clients and therapists compared to conventional interventions. Practical and logistical factors such as the significant cost as well as the space and training required for equipment operation are pressing influences on therapists considering the use of VEs in clinical practice [92, 93]. The reviewed findings provide therapists with additional information to consider beyond these practical realities when deciding what type of VE might be best suited for their needs. Specifically, therapists should begin by closely observing the quality of patient movement in VEs, considering the differences in how individuals with impairments move in flat-screen VEs or HMDs as compared to in real environments. This is particularly relevant when the goal is to eventually integrate these environments into unsupervised home-based practice. However, more research is required to determine the clinical significance of these differences. Such observations can guide decisions to use verbal feedback, demonstration or physical guidance to encourage movements that are relevant to real-world activities. These strategies can be used to explicitly emphasize transfer within VE-based interventions; for example, by combining VE practice of a part-task component with practice of the ‘whole’ task in the real world. Further, following Quadrado’s earlier suggestion, virtual tasks should be more challenging that the corresponding real-world task to support transfer [69]. While this is still speculation, therapists may consider increasing the challenge in VE practice by taking advantage of VE attributes; for example, by adding cognitive dual-task challenges with either visual or auditory modalities. Lower-fidelity VEs may be more realistic options for patients with significant physical or cognitive limitations. However, it is clear that decisions about type of VE display and interaction method should be made in consideration of patient goals, abilities and nature of the practice setting, including the availability of patient supervision and monitoring.

#### Next steps for research

More basic and clinical studies should evaluate the impact of differing kinematics and learning mechanisms between virtual and real environments over longer timescales and on transfer outcomes [81]. While fully replicating reality in VEs is unattainable and undesired, it is critical to determine which perceptual, cognitive and motor attributes of VEs are essential to enhance transfer and generalization [86]. In particular, further work should aim to understand whether inherent differences in haptic input are limiting factors for transfer. HMDs are becoming more clinically accessible; determining the advantages with respect to skill acquisition and transfer of these viewing mediums over flat-screen display VEs is required. This is especially important as the fidelity benefits of HMDs must be balanced with possible physical risks posed by prolonged interaction, including visual strain, [94] motion sickness [95] and postural imbalance [96].

To date, the VE training strategies (reviewed in Section 1) that emphasize measuring or manipulating variability have not been explored in the context of transfer from virtual to real environments. This emphasis on the role of variability in VE skill learning may have transfer relevance. Practice that includes multiple task variations may support the learner’s ability to transfer skill to unpracticed contexts. VEs offer the potential to vary task presentation in more fine-grained detail than what is possible in the real world. However, to the authors’ knowledge, little research has explored whether inducing variability in VEs enhances transfer to real-world tasks. Clearly, such variability should be a key characteristic of the real-life task and therefore, likely to be relevant for transfer. In addition, given the differing sensory-motor information between virtual and real environments, the extent to which practice in VEs may elicit more movement variability as compared to that elicited by practice in real environments is open for exploration. We advocate for this type of investigation, because the redundancy inherent to complex skills naturally invites variability, and VEs are ideal testbeds to measure and evaluate it. Overall, much remains to be learned about how VE affordances might facilitate or limit learners’ exploration of the solution space, and whether such exploration might enhance transfer to real-world performance.

## Considerations and conclusions

### Challenges of virtual environments as experimental tools

Alongside the numerous advantages, VE experimental platforms can also present many technological challenges. For instance, the considerable cost, space requirements, and programming expertise required to develop and operate custom applications in specialized VEs with multiple data collection peripherals (e.g. motion capture cameras, haptic gloves, inertial measurement units, or external stimulus triggering) can be prohibitive. While off-the-shelf software and hardware can be low in cost, they may not be sufficiently customizable or suitable for rehabilitation populations. Researchers who use technologies originally designed for entertainment and gaming must take the additional steps of validating the precision and accuracy of the equipment to meet clinical standards, a task undertaken in the time before an updated version is released or the technology becomes obsolete. The availability of open-source and source-available gaming engines (e.g., Unity and Unreal Engine), 3D graphics/animation software (e.g., Blender), and microcontroller software and hardware (e.g., Arduino) has dramatically increased over the last decade. This has made it easier and more affordable for developers to learn and use these tools. However, the learning curve remains steep. In particular, care is needed to avoid both the sensory conflict that elicits motion sickness [97] and the ‘uncanny valley’, a term that describes the discomfort of seeing simulations designed to look human, but that fall short of natural human looks and behavior [98]. Ultimately, from a clinical viewpoint, it is certainly more practical to ask a patient to practice a task in the real world as compared to the time, energy and financial resources required to render the task virtually. Customized VE platforms that are already designed for rehabilitation reduce this burden by providing turn-key clinically-relevant assessments, but these systems are still more costly than off-the-shelf options.

### Opportunities of virtual environments

Despite these concerns, VEs remain powerful research platforms to evaluate motor learning of complex skills and develop training strategies to facilitate learning. They are also effective rehabilitation interventions, whose impact will be strengthened by greater understanding of the relationships between viewing medium, interaction fidelity and virtual rendering with skill transfer from virtual to real environments. We argue that a focus on studying complex tasks with nested redundancy is required to advance both of these research interests. These two domains – basic science and clinical implementation - can be combined through a call for researchers to move from simple to complex skills in VEs, taking advantage of understanding and controlling the task physics to measure and manipulate the variability inherent in learning.

The goal of this review was to summarize insights from studies on complex tasks in VEs that illuminate the role of movement variability for learning and discuss options for VEs to manipulate task attributes to provide novel forms of feedback and guidance. We summarized the current state of knowledge about transfer from VEs to the real world that emphasized how much still needs to be understood: which perceptual, cognitive and motor features of real-world tasks and behaviors must be components of VEs for transfer to occur? We have identified broad areas where more research is required; however, we did not systematically appraise study quality, and subsequent reviews should do so to support further clinical recommendations. This program of research is significant: it can inform clinical decision-making about how best to apply VEs in rehabilitation and identify the virtual task delivery and presentation conditions required to improve skill transfer from VEs to the real world.

## Data Availability

Not applicable

## Abbreviations

2D: Two-dimensional
3D: Three-dimensional
CP: Cerebral palsy
DMD: Duchenne muscular dystrophy
HMD: Head-mounted display
VE: Virtual environments
